# Tissue Necrosis due to Chloroform: A Case Report

**Published:** 2013-10-07

**Authors:** Nahid Mohammadzadeh Akhlaghi, Ladan Baradaran Mohajeri, Mahta Fazlyab

**Affiliations:** a Department of Endodontics, Dental Branch, Islamic Azad University, Tehran, Iran*;*; b Dental Research Center, Research Institute of Dental Sciences, Shahid Beheshti University of Medical Sciences, Tehran, Iran

**Keywords:** Chloroform, Extrusion, Tissue Necrosis, Retreatment

## Abstract

For many years, gutta-percha has been the root canal filling material of choice. Chloroform is one of the most efficient solvents widely used for gutta-percha removal in retreatment cases, despite being toxic and carcinogenic. The present case report discusses a chloroform extrusion through an existing perforation to the surrounding periodontal ligament space and subsequent necrosis in supporting bone and tissues, during an endodontic retreatment visit for an addicted patient. Subsequently, the management and preventive options are reviewed.

## Introduction

Dental materials are frequently placed in direct contact with live tissues and endodontic materials are not an exception [1]. Endodontic retreatment is often the preferred course of action for failed root canal therapy [2-6]. Gutta-percha (GP) has been the most frequent root canal obturation material for more than 100 years [3, 4, 6] which is simple enough to remove in case of endodontic failure. There are several methods for GP removal: thermal, mechanical and chemical solvent [4, 6]. Chloroform is one of these inorganic solvents widely used for GP softening or dissolving [4]. With less solving ability, Xylol is another GP solvent followed by essential oils *i.e*. Eucalyptol and orange oil [7, 8].

The efficiency, safety and benefit of chloroform as a solvent for softening GP during endodontic treatment has been proven [5]. In an attempt to assess the antimicrobial effect of chloroform on *Enterococcus faecalis*, Edgar *et al.* assumed that chloroform reduced bacteria to non cultivable levels [5]. However, side effects from exposure to chloroform have also been recorded [1]. Furthermore, studies have addressed that chloroform is possibly carcinogenic to humans [6, 9]. Chloroform is classified as a group 2B carcinogen by the International Agency for Research on Cancer, which points out the materials that lack adequate evidence of carcinogenicity in humans but, there is sufficient evidence of their carcinogenicity in animals [4].

This case represents over extrusion of chloroform through a perforated tooth that caused vast alveolar and gingival necrosis.

## Case Report

A 46 year old man with the history of drug abuse for the last 15 years without any systemic disease was referred to our office by a general practitioner. The dentist stated that he was doing a root canal treatment (RCT) for upper central and lateral incisors and canine without being aware of the occurrence of a perforation during access cavity preparation on lateral teeth. He prepared and obturated the canals. After providing the final radiography he decided to extirpate the GP from PDL space without anesthesia, by means of Chloroform, again being unaware of its leakage through the perforation site. The patient did not feel any pain neither unpleasant feeling which was believed to be due to his addiction. He was dismissed and the following morning he came up with a large missing zone in his buccal gingiva. He was immediately referred by his dentist to an endodontist for problem solving.

**Figure 1:**
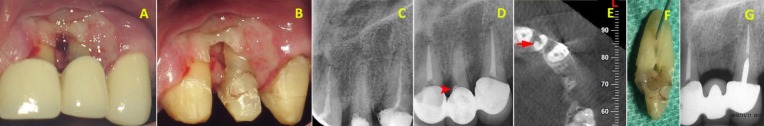
Tissue necrosis on maxillary left lateral incisor after two *A)* and ten *B)* days of injury; *C)* initial x-ray of the maxillary left lateral incisor; *D)* post-endodontic x-ray shows lateral perforation (red arrow head), along the mesial wall of the root in the lateral incisor; *E)* the CBCT shows the perforation in the mesial wall (red arrow); *F)* the gross view of perforation in the extracted tooth and; *G)* six-month follow-up

A large necrotic area in the gingival areas surrounding upper lateral incisor was obvious during clinical examination (Figures 1A and 1B). Lateral incisor had a large longitudinal perforation on mesial wall of the root. The patient reported history of drug abuse and that he had felt no pain or irritation during the retreatment. He also reported that he has experienced a medical surgery without anesthesia years before. After consulting with a periodontist, nonsurgical retreatment of upper central incisor and canine was performed by the endodontist (Figures 1C and 1D). Before treatment, the patient was referred for Cone Beam Computed Tomography (CBCT) for more evaluation that showed a long perforation on coronal half of mesial wall of maxillary left lateral incisor (Figure 1E).

The lateral incisor was extracted and during a flap surgery the area was re-contoured. The extracted tooth showed a longitudinal perforation (Figure 1F). After the time needed for gingival healing, the patient treatment was completed with fixed partial denture between central incisor and the canine. Figure 1G shows the six-month follow-up.

## Discussion

This article reported a case in which the necrosis following lateral extrusion of chloroform during retreatment led to necrosis of the surrounding tissues and tooth loss.

In case of perforation during access cavity preparation, maintaining the endodontic irrigants and solvents within the limitation of canal, seems of utmost importance [10]. This is especially true during retreatment, as residual chloroform remains for a certain time in dissolved GP and the toxicity of mixture is sustained. Chloroform is able to bind to cell membrane and readily penetrate the cells leading to lethal cell injury [9]. Vajrabhaya *et al.* found that the percentage of cells viability after contact with Chloroform was around 5-6% [4]. Their results provided enough information to dentists to warn of any GP solvent discharge through apical foramen [4].

Metzenger and Ben Amar proposed a procedure for the removal of an over extended root filling in 2 steps [10]. The upper part of GP was removed by a solvent and then the depth of 3 mm short of the apex was removed by a Headstrom file. In this way they prevented over flow of chloroform out of the canal. By dissolving rather than softening, chloroform leaves residues on the canal and pulp chamber walls and due to fast evaporation, it needs to be refreshed. On the other hand, in controlling and removing softened rather than the liquefied GP, xylol proves to be a more efficient and biologically safer solvent [11, 12]. Cytotoxicity data have shown that chloroform was able to produce cellular death in a dose related fashion, the strongest effect being observed at higher concentrations [9]. Apical extrusion of debris produced in endodontic (re)treatment might lead to post operative pain and discomfort [12].

## Conclusion

In conclusion, chloroform is a strong cytotoxicant, the exposure to which is of special concern.
